# Forensic Aspects of a Fatal Intoxication Involving Acetaminophen, Citalopram and Trazodone: A Case Report

**DOI:** 10.3390/toxics10080486

**Published:** 2022-08-22

**Authors:** Giulio Mannocchi, Roberta Tittarelli, Flaminia Pantano, Francesca Vernich, Margherita Pallocci, Pierluigi Passalacqua, Michele Treglia, Luigi Tonino Marsella

**Affiliations:** 1 School of Law, University of Camerino, Piazza Cavour 19/f, 62032 Camerino, Italy; 2 Section of Legal Medicine, Social Security and Forensic Toxicology, Department of Biomedicine and Prevention, University of Rome Tor Vergata, Via Montpellier 1, 00133 Rome, Italy; 3 Independent Researcher, Via della Divisione Torino 69, 00143 Rome, Italy

**Keywords:** acetaminophen, citalopram, trazodone, poly-drug intoxication, dog attack, gas chromatography-mass spectrometry

## Abstract

We report the case of a young man, a former heroin addict, found dead at home by the Police Forces in an advanced state of decomposition. Numerous blisters and unpacked tablets of medications were found all over the bed and on the floor of the room. Multiple injuries to the face, left arm and neck of the deceased were noted. The latter damages were attributed to post-mortem dog bites, since no indications of a possible defense against the animal were observed. The autopsy findings were unremarkable. Toxicological investigations performed on peripheral blood and urine by gas chromatography-mass spectrometry (GC-MS) technique showed the presence of acetaminophen, citalopram and trazodone. Combined drug intoxication was proposed as the cause of death since acetaminophen and trazodone concentrations were comparable with the ones found in fatal cases. Moreover, citalopram concentration in peripheral blood was above the toxic range and in accordance with levels found in fatalities due to poly-drug intoxication.

## 1. Introduction

### 1.1. Acetaminophen

Acetaminophen, also known as paracetamol, is a common pain reliever and antipyretic used since 1955 for the management of several pathological conditions such as headache, osteoarthritis, chronic backache and postoperative pain as part of a multimodal approach [1]. It is commercially available alone or in combination with other drugs [2]. In Italy, paracetamol in specific formulations and only at certain dosages can be sold as an over-the-counter drug (without a prescription). Paracetamol is a safe drug when administered at therapeutic doses; however, several fatal cases related to the onset of hepatotoxicity consequent to the administration of single or repeated high doses and chronic ingestion were reported, starting from the mid-1980s [3]. Adverse reactions commonly related to paracetamol intoxication are acute liver failure (ALF), centrilobular hepatic necrosis, hypoglycaemic coma and renal tubular necrosis [4,5]. Liver damage is caused by the excessive production of its toxic breakdown metabolite, N-acetyl-p-benzoquinone imine (NAPQI). At therapeutic doses, approximately 5–9% of acetaminophen is converted by Cytochrome P 450 (CYP 2E1, CYP1A2 and CYP 3A4) to NAPQI [6]. Normally, it is inactivated in conjugation with the sulphydryl groups of glutathione, but in overdose cases, glutathione is depleted, and NAPQI is not detoxified. The exact mechanism by which toxicity occurs is not known, but it has been proposed that in the case of GSH depletion, NAPQI causes toxicity by binding to cellular macromolecules. Treatment for acetaminophen intoxication is based on the administration of N-acetylcysteine, which is associated with mortality reduction of less than 30% [6].

Paracetamol overdoses are frequently related to suicide intents, but cumulative or accidental ingestion is also reported. Recently, unconventional uses of paracetamol for the treatment of sleep disorders, to enhance athletic performances and mixed with drinks, waterpipe and illicit drugs were reported by Bloukh et al., especially among patients with a history of substance use, parents of young children or athletes [7].

Several acetaminophen intoxications are due to the co-products contained in paracetamol formulations, such as caffeine, dextromethorphan, codeine, oxycodone, antihistamines, acetylsalicylic acid and propoxyphene [8]. Furthermore, several contributing factors could be implicated in the induction of paracetamol hepatotoxicity even at therapeutic doses: malnutrition, alcohol abuse and liver impairment [9]. Therapeutic blood concentrations range from 10 to 25 mg/L, whereas toxic and lethal values range from 100 to 150 mg/L and 200 to 300 mg/L, respectively [10]. However, paracetamol hepatotoxicity is known to be dose-dependent, different from the toxic liver damage induced by the use of other drugs or herbs, which can be idiosyncratic [11,12].

### 1.2. Citalopram

Citalopram is an antidepressant drug, only available by prescription, belonging to the selective serotonin reuptake inhibitor (SSRI) class, approved for the treatment of major depression and panic disorders with or without agoraphobia with minimal effects on norepinephrine and dopamine reuptake [13]. In the case of therapeutic use, it is rarely associated with severe side effects and serotonin syndrome (SS), characterized by specific symptoms such as mental status changes, autonomic hyperactivity and neuromuscular abnormalities, occurs only in a few cases, often related to alterations in cytochrome P450 (CYP)-mediated metabolism [14]. Recreational uses of citalopram are not currently reported in the literature. Fatalities attributed either to citalopram alone or in combination with other drugs have been reported in the scientific literature [13,14,15,16,17,18]. Therapeutic oral doses range from 20 to 40 mg per day, with a maximum daily dose of 60 mg, and therapeutic concentrations in the blood range from 0.02 to 0.2 mg/L. The lethal concentration is 0.5 mg/L [19]. Other authors suggest the following therapeutic, toxic and comatose/fatal values: 0.05–0.11 mg/L, from 0.22 mg/L and from 5–6 mg/L, respectively [10].

### 1.3. Trazodone

Trazodone is a psychoactive substance, purchasable by prescription only and belonging to the piperazines class, approved by the Food and Drug Administration (FDA) for the treatment of depression [20]. However, several off-label uses of this compound are reported. This drug is also prescribed for the treatment of insomnia, bulimia, anxiety disorders, alcohol and benzodiazepine dependence, degenerative diseases of the central nervous system, fibromyalgia, sexual dysfunction, chronic pain and schizophrenia [21]. Its mechanism of action includes serotonin (5-HT-5-hydroxytryptamine) uptake inhibition, but the strongest effect develops through antagonism towards 5-HT2/1C receptors [22]. Some fatality reports due to trazodone intake, alone or in combination with other substances, have been described in the literature [23,24,25,26]_._ Therapeutic and toxic concentrations in the blood range from 0.7 to 1 mg/L and from 1.2 to 3–4 mg/L, respectively, whereas the comatose–fatal concentration has been suggested to be 12–15 mg/L [10].

Recreational use of trazodone is increasing. Trazodone is sold in the illicit market under the street name “sleepeasy” for its relaxing and calming effects: this drug is commonly taken by snorting or smoking the crushed tablets mixed with marijuana or by adding trazodone powder to alcohol. Concomitant use of trazodone with other substances, such as alcohol, ecstasy or methamphetamine, enhances its effects. These routes of administration expose users to a high risk of overdose and other harmful side effects. In cases of misuse, dependency and addiction can also occur.

## 2. Case Report

### 2.1. Scene of Death Inspection

We report the case of a Caucasian man, 32 years old, found dead in the bedroom of his flat by the Police Forces. The intervention of law enforcement was requested by the father of the deceased, as he had not heard from him for about 15–20 days. He was initially unconcerned about his silence because of a previous family quarrel, but due to the unanswered doorbell and insistent barking of the dog from the apartment, the relatives alerted the fire department. The apartment door was locked from the inside, with a key stuck in the lock. Criminal evidence of violence was missing.

The victim was found in the bedroom, dressed in his shoes and with the upper half of the body tucked under the left side of the bed. From the information provided by law enforcement officers who attended the scene, the deceased was a former heroin user. His doctor was unaware of his addiction history, and it is not known whether the drugs found in his apartment had been illegally obtained.

**Figure 1:**
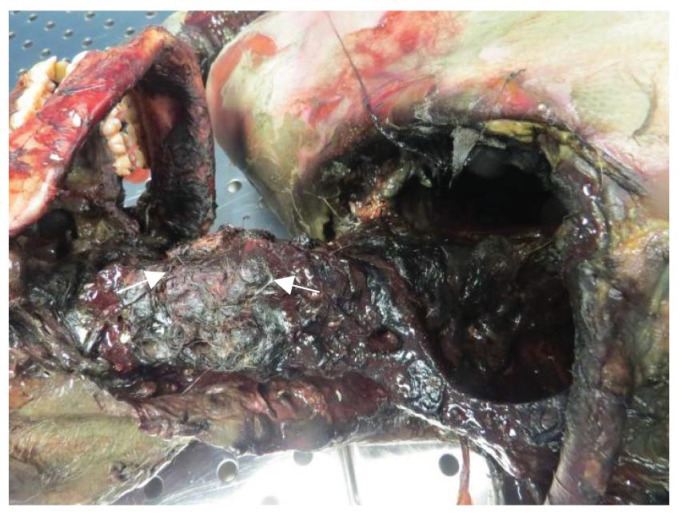
Skeletonized skull and cervical spine tract. Dog hair is noticeable on the neck (white arrows).

Numerous blisters and unpacked tablets of medications were found all over the bed and on the floor of the room. The forensic pathologist who was called at the scene excluded criminal action as the cause of death since there were no signs of forced entry and no evidence to suggest pre-fatal external injurious action. Multiple injuries to the face, left arm and neck were noted. In particular, the soft tissues of the face and neck were completely absent, exposing the underlying bone structures. (Figure 1). The latter damages were attributed to post-mortem dog bites since no indications of a possible defence against the animal were observed. The dog that was found in the flat of the deceased had torn and fed on the soft parts of the dead body. The rest of the body was clothed and the right arm, covered by a blanket, was not ripped.

### 2.2. Autopsy Findings

The deceased was an Italian male in an advanced state of decomposition. Because of the alterations caused by putrefactive phenomena, the forensic pathologist estimated that the post-mortem interval ranged from 10 to 15 days. External examination revealed nothing remarkable. The head and the neck were completely skeletonized without fractures. The left arm consisted exclusively of the humerus. A part of the forearm and elbow soft tissues and the fingers of his left hand were missing due to the dog’s action. Another injury was noticed in the poster lateral trunk, also caused by the dog. There was no trauma to the lower extremities. The internal examination showed congestion signs in the examined organs. There were no fractures to the skull base, and there was no evidence of hemorrhage involving the brain and the soft tissues of the chest. The internal organs were removed from the chest cavity due to the dog’s action. The organs of the abdominal cavity, though in an advanced state of putrefaction, did not show any traumatic injury. There was no pre-existing disease that could cause the death. Moreover, since the deceased was known to be a former heroin user, as reported by the victim’s relatives to the Police Forces, peripheral blood and urine specimens were collected for toxicological analysis.

### 2.3. Histological Findings

**Figure 2:**
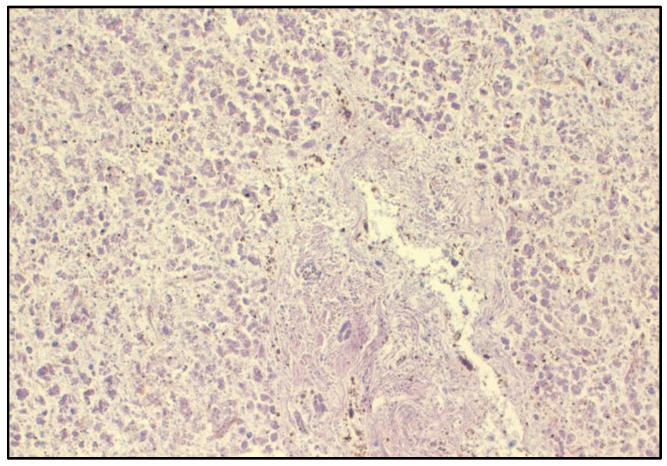
Histological examination of hepatic tissue. Magnification 40×. Hematoxylin and eosin stain.

Sections of splenic, hepatic, encephalic and renal tissues were investigated to determine the presence of morphological and functional alterations. All the slides were analyzed with the hematoxylin and eosin (H&E) staining technique. The histological examination performed on splenic tissue showed the presence of thanatological changes due to the advanced state of decomposition. The capsule was regular; in splenic tissue, macrophages with pigmented cytoplasmic granules were detected. A preserved lobular architecture was observed in the liver section. Hepatocytes were poorly preserved because of post-mortem alterations. Chronic cholestatic liver disease and mild fibrosis (L1) in the portal area were noticed (Figure 2). Encephalic slides revealed the presence of cerebral edema, particularly in perivascular spaces, without traumatic signs. Histological observation of kidney sections showed serious thanatological modifications with glomerular and tubular shadows.

## 3. Materials and Methods

### 3.1. Samples

Toxicological analyses were performed on unpreserved peripheral blood (35 mL) and urine (40 mL), collected during the autopsy and stored at −20 °C until the analysis.

### 3.2. Chemical and Reagents

Reference standards for paracetamol, paracetamol-*d*4 (IS), citalopram, trazodone and methadone-*d*9 (IS) were purchased from Cerilliant^®^ (Round Rock, TX, USA). Ethyl-acetate from Carlo Erba^®^ (Milan, Italy) and BSTFA (with 1% TMCS) were purchased from Sigma-Aldrich (Milan, Italy). Ultrapure deionized water was homemade (Millipore^®^ Helix 70).

### 3.3. Qualitative Analysis

**Figure 3:**
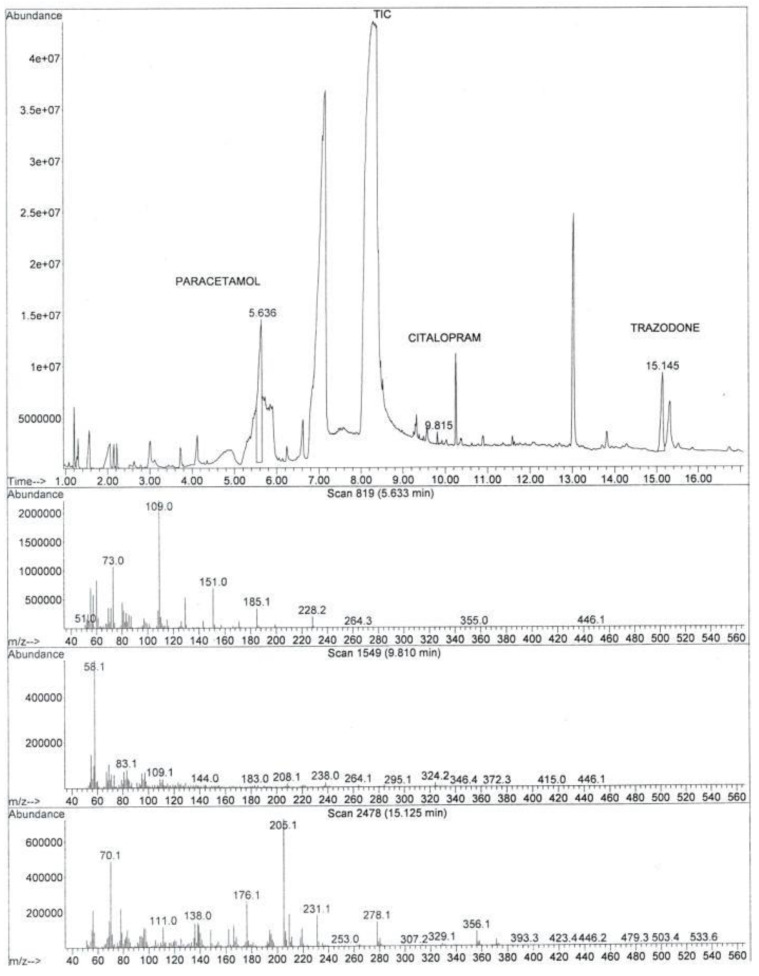
Full scan mode chromatogram and mass spectra obtained from peripheral blood analysis.

Immunochemical screening (ILab 650, Instrumentation Laboratory, Milan, Italy) was performed on the urine sample with a positive result for amphetamines and MDMA (3,4-methylenedioxymethamphetamine). The toxicological screening performed on urine was negative for opiates, methadone, cannabinoids, cocaine, benzodiazepines and alcohol. The positive amphetamines and MDMA immunoassay results were not confirmed by the analysis in gas chromatography coupled with mass spectrometry (GC/MS). The false positive results were probably related to the presence of trazodone [27]. As part of routine investigation, aliquots of non-diluted peripheral blood and urine were analyzed using a screening method in GC-MS (Figure 3). Samples were extracted at pH 8.0 (adding 50 mg of solid HCO3-/CO3-- buffer) with 4 mL of ethyl acetate after 15 min stirring. After centrifugation (4000 rpm, 3 min), the organic layer was evaporated to dryness under a gentle stream of nitrogen. The residues were reconstituted in 50 µL of ethyl acetate (Figure 3).

### 3.4. Quantitative Analysis of Acetaminophen

To 100 µL of diluted (1:100) fortified blank urine, peripheral blood and samples, 200 ng of paracetamol-*d*_4_ were added. Samples were then extracted at pH 4.0 (adding 500 µL of acetate buffer) with 4 mL of ethyl acetate after 15 min stirring. After centrifugation (4000 rpm, 3 min), the organic layer was collected and evaporated to dryness under a gentle stream of nitrogen. The residues were derivatized using 50 µL of BSTFA+1% TMCS and put in a heating block for 30 min at 70 °C.

### 3.5. Quantitative Analysis of Citalopram and Trazodone

To 100 µL of diluted (1:100) fortified blank urine, peripheral blood and samples, 100 ng of methadone-*d*9 were added. Samples were extracted at pH 8.0 (adding 50 mg of solid HCO3--/CO3-- buffer) with 4 mL of ethyl acetate after 15 min stirring. After centrifugation (4000 rpm, 3 min), the organic layer was evaporated to dryness under a gentle stream of nitrogen. The residue was reconstituted in 50 µL of ethyl acetate.

### 3.6. Instrumentation and Conditions

GC analysis was carried out on a gas chromatography instrument Agilent HP 7028A GC coupled with an Agilent MSD 5975. The capillary column used was an HP-5MS (17 m × 0.25 mm I.D coated with a 0.25 µm film). The GC conditions were as follows: the column temperature was programmed from 120 °C to 290 °C with an increase of 15 °C/min; the injection port and the transfer line temperature was 270 °C; helium was used as carrier gas at a flow rate of 1ml/min; split ratio 10:1. The mass analyzer was operated by electron impact (70 eV) in full scan mode for qualitative analysis (mass range 40–500 *m*/*z*).

For quantitative analysis, the mass analyzer was operated by electron impact (70 eV) in selected ion monitoring (SIM). Quantitative analysis of paracetamol was carried out recording ions *m*/*z* 206-280-295 for paracetamol and *m/z* 299 for paracetamol-*d*4. The underlined ions were used for quantitative analysis (target/qualifier). Quantitative analysis of trazodone and citalopram was performed, recording ions *m*/*z* 205-278-356 for trazodone, *m*/*z* 58-324-238 for citalopram and *m*/*z* 78-229-303 for methadone-*d*9. 

Validation of new analytical methods to be used in a single case study or for the analysis of rare analytes was performed for blood and urine, in accordance with updated established international criteria [28]. 

The following parameters were evaluated for a quantitative method: selectivity, linearity, accuracy, precision, the limit of detection (LOD) and lower limit of quantification (LLOQ). Selectivity was evaluated by checking the interfering signals of blank matrices and interfering signals of paracetamol-d_4_ and methadone-d_9_ through zero samples. The standard acetaminophen, citalopram and trazodone calibration curves were obtained by fortification of blank human blood (5 levels) and blank human urine (5 levels) with an appropriate amount of pure standards in a range of concentration from 50 to 800 μg/mL for acetaminophen and trazodone and from 2.5 to 40 μg/mL for citalopram.

Blood and urine methods were linear for all the analytes, with a determination coefficient (R2) ranging from 0.991 to 0.997. The precision for all the analytes was always lower than 15% (CV%), while bias never exceeded ±15%.

The limit of detection (LOD) for peripheral blood was 4.80 μg/mL for acetaminophen, 0.34 μg/mL for citalopram and 0.28 μg/mL for trazodone with a signal-to-noise ratio (S/N) of 3. The latter values fit the purpose of the current case.

LOD and LLOQ evaluated for blood and urine are reported in Table 1. 

## 4. Results

Acetaminophen, citalopram and trazodone were detected in peripheral blood and urine using a non-targeted analysis performed by GC-MS. Blood alcohol and volatile compounds determination were carried out by gas chromatography with a head-space FID detector (HS-GC-FID), and alcohol was detected at 0.47 g/L. The alcohol concentration found in peripheral blood, may be related with the advanced state of decomposition of the body. Several authors correlate an increase in the alcohol concentration with post-mortem production due to the action of different species of bacteria. In most of the cases in which the production of post-mortem ethanol was observed, the concentration was not higher than 0.3 g/L [29,30,31,32]. In Table 2, the analyte concentrations found in peripheral blood and urine are reported. To date, it is not known to what extent the advanced state of decomposition affected the concentration of the three analytes detected.

## 5. Discussion

To the best of our knowledge, a fatal case involving all three of the substances found here has not been described before in the literature. Nevertheless, fatalities attributed to intoxication by acetaminophen together with citalopram are documented. Moore et al. reported a fatal case due to metaxalone and gabapentin intoxication, in which acetaminophen and citalopram were detected together with the latter compounds in heart blood at the concentrations of 97 mg/L and 0.4 mg/L, respectively [16]. Seetohul et al. presented a post-mortem case involving citalopram and acetaminophen, among other substances: nefopam, nicotine, caffeine, amitriptyline, gabapentin and diazepam. The death was attributed to atherosclerotic coronary artery disease and therapeutic drug toxicity. Citalopram was quantified at 0.7 mg/L and 0.9 mg/L in unpreserved femoral and cardiac blood, respectively, whereas acetaminophen concentrations were not provided [33]. Acetaminophen and citalopram are among the 10 most frequently detected drugs in hanging and drug poisoning (intoxication) suicides. Moreover, poly-drug use was found to be more common in intoxication suicides, 3.6 drugs/case compared with 1.8 drugs/case in hanging cases. Acetaminophen and citalopram were detected in femoral blood in 652 and 345 out of 2468 intoxication suicides at the median concentration of 20 and 0.7 mg/L, respectively. Similarly, citalopram and acetaminophen were found at the median concentration of 0.3 and 5 mg/L in 428 and 396 out of 4551 hanging suicide cases [34]. Therapeutic, toxic and fatal blood concentrations, as presented by Schulz et al. for the three substances detected in the presented case, are reported in Table 3 [10].

In relation to trazodone toxicity, a few cases of attributable deaths have been reported in the literature, with both cardiovascular and liver toxicity occurring. In the latter cases, both an idiosyncratic reaction [35] and mediated by mechanisms of acute cytotoxicity and cholestasis have been shown. In a study carried out to test the molecular mechanisms underlying trazodone toxicity, it was shown that exposure to the drug results in increased lipid peroxidation with increased production of ROS (Reactive Oxygen Species), as well as reduced cellular GSH content [36].

With reference to the case under investigation, from a pathophysiological point of view, it is possible that a negative synergistic action between trazodone and acetaminophen occurred; in this context, trazodone caused, among other effects, an increase in cellular ROS production and a reduction in GSH reserves, thus exacerbating the toxicity of NAPQI, which, in cases of GSH depletion, is not inactivated, making its toxicity particularly evident, especially in the liver. Concerning the interaction between trazodone and other psychoactive drugs, in a study conducted in Italy in 2020 on 97 patients with depressive syndrome, the interaction between trazodone, citalopram and fluoxetine was studied over a period of 1 year. The results of this study showed that the use of citalopram and fluoxetine in combination with trazodone did not have a significant impact on serum trazodone concentrations; furthermore, no cases of headache, daytime sedation, fatigue or serotonergic syndrome were reported during the study [37].

In relation to the stability of the investigated substances in biological samples collected from decomposed corpses, there are notmuch data available in the literature. 

Karinen et al. measured concentrations of acetaminophen in forensic blood specimens after long-term storage (16–18 years) at −20 °C. The authors reanalyzed 13 blood samples, and in 10 of these, the results were within ±30% of the initial concentrations, and they concluded that generally, the results decreased slightly than to those reported by other authors [38,39].

Citalopram stability was studied by Moretti et al. in dried blood spots (DBSs) stored at room temperature for three months. DBSs were analyzed in triplicate immediately after collection, within the following 3 weeks and after 3 months. The long-term stability (3 months) was also tested in blood specimens stored at −20 °C. The authors observed that citalopram stability in DBSs was about 2–3 weeks, and there was also degradation in blood specimens by more than 50% after 3 months of storage [40]. Their findings were comparable to those previously reported by other authors: Lewis et al. reported stability of 5 days for citalopram in whole blood specimens stored at 4 °C, whereas Karinen et al. reported a stability up to 1 year in post-mortem blood samples after storage at −20 °C [41].

Martin et al. studied the tissue distribution and post-mortem redistribution of trazodone in two fatalities, and they observed that trazodone had low potential for post-mortem redistribution lacking significant solid organ deposits of the drug. The authors also reported that trazodone concentrations were relatively stable in post-mortem blood samples, with a lower increase than 40% observed 60 h after the death and a less than two-fold change during early putrefaction. For peripheral blood samples, they observed marked stability with no significant change [42].

McIntyre et al. compared trazodone concentrations in liver, peripheral blood and central blood in 19 medical examiner cases stored up to eight months. The authors observed a minor degradation of trazodone in post-mortem blood samples stored at 4 °C and about a 20% decrease in samples stored up to eight months. The data collected by the authors demonstrated that trazodone was unlikely to show significant redistribution [43].

## 6. Conclusions

Combined drug intoxication was proposed as a cause of death since acetaminophen and trazodone concentrations were comparable with the ones found in fatal cases. Moreover, citalopram concentration in peripheral blood was above the toxic range and in accordance with levels found in fatalities due to poly-drug intoxication. From a pathophysiological point of view, based on the results of the toxicological and autopsy examinations performed (particularly histological ones), it is possible to ascribe the death to acute liver failure due to a synergistic action between drugs (acetaminophen and trazodone in particular).

## Figures and Tables

**Table 1:** LOD and LLOQ for acetaminophen, trazodone and citalopram in blood and urine.

	Blood		Urine	
Compound	LOD (µg/mL)	LLOQ (µg/mL)	LOD (µg/mL)	LLOQ (µg/mL)
Acetaminophen	4.80	15.00	2.50	8.10
Citalopram	0.34	1.10	0.10	0.32
Trazodone	0.28	0.90	0.18	0.60

**Table 2:** Analytical results in biological matrices.

Samples	Acetaminophen (μg/mL)	Citalopram (μg /mL)	Trazodone (μg/mL)
Peripheral Blood	328	2.7	21
Urine	155	50	109

**Table 3:** Therapeutic, toxic and fatal blood concentrations as provided by Schulz et al. [10].

Substance	Blood-Plasma Concentration (μg/mL)		
	Therapeutic (“Normal”)	Toxic (From)	Comatose-Fatal (From)
Acetaminophen	(5-)10–25	100–150	200–300
Citalopram	0.05–0.11	0.22	5–6
Trazodone	0.7–1	1.2; 3–4	12–15

## Data Availability

Not applicable.
